# Discovery of ABBV-4083, a novel analog of Tylosin A that has potent anti-*Wolbachia* and anti-filarial activity

**DOI:** 10.1371/journal.pntd.0007159

**Published:** 2019-02-28

**Authors:** Thomas W. von Geldern, Howard E. Morton, Rick F. Clark, Brian S. Brown, Kelly L. Johnston, Louise Ford, Sabine Specht, Robert A. Carr, Deanne F. Stolarik, Junli Ma, Matthew J. Rieser, Dominique Struever, Stefan J. Frohberger, Marianne Koschel, Alexandra Ehrens, Joseph D. Turner, Marc P. Hübner, Achim Hoerauf, Mark J. Taylor, Stephen A. Ward, Kennan Marsh, Dale J. Kempf

**Affiliations:** 1 Global Pharmaceutical Research and Development, AbbVie, North Chicago, Illinois, United States of America; 2 Franciscan Institute for World Health, Franciscan University, Steubenville, Ohio, United States of America; 3 Centre for Drugs and Diagnostics, Department of Parasitology, Liverpool School of Tropical Medicine, Liverpool, United Kingdom; 4 Institute for Medical Microbiology, Immunology and Parasitology, University Hospital, Bonn, Germany; McGill University, CANADA

## Abstract

There is a significant need for improved treatments for onchocerciasis and lymphatic filariasis, diseases caused by filarial worm infection. In particular, an agent able to selectively kill adult worms (macrofilaricide) would be expected to substantially augment the benefits of mass drug administration (MDA) with current microfilaricides, and to provide a solution to treatment of onchocerciasis / loiasis co-infection, where MDA is restricted. We have identified a novel macrofilaricidal agent, Tylosin A (TylA), which acts by targeting the worm-symbiont *Wolbachia* bacterium. Chemical modification of TylA leads to improvements in anti-*Wolbachia* activity and oral pharmacokinetic properties; an optimized analog (ABBV-4083) has been selected for clinical evaluation.

## Author summary

The *Wolbachia* bacterium lives symbiotically within the filarial worms that cause onchocerciasis and lymphatic filariasis. In the absence of these bacteria juvenile worms cannot mature, females are unable to reproduce, and the worm life-span is significantly shortened. Thus, anti-*Wolbachia* therapy would seem to be an ideal approach to treating filarial disease. This concept has been validated clinically using the tetracycline antibiotic doxycycline. However, doxycycline, which is contraindicated in children and women of child-bearing age, is not ideal for field use. Additionally doxycycline requires a long course of treatment (minimum 4 weeks of daily use) to provide clinical benefit. A safer, faster anti-*Wolbachia* agent would be a valuable addition to the filariasis pharmacopeia. Through targeted screening, we have identified the veterinary antibiotic Tylosin A (TylA) as an effective anti-*Wolbachia* lead compound. While the *in vitro* and *in vivo* activity of TylA match that of doxycycline, its potency is not ideal, and it suffers from poor oral bioavailability. By appropriate derivatization of the 4”-OH group of TylA (on the mycaminose sugar) we were able to improve oral absorption while simultaneously increasing anti-*Wolbachia* potency. Optimization of this substituent with a focus on metabolic stability led to the identification of ABBV-4083, the 4”-(4F-benzyl) analog. ABBV-4083 is exquisitely active against *Wolbachia*, with an improved pharmacokinetic profile. This analog outperforms doxycycline in several animal models of filariasis; in particular it clears the bacteria effectively with substantially shortened dosing regimens. Preclinical efficacy and safety studies indicate that ABBV-4083 may have a desirable profile as a novel, next-generation anti-filarial agent.

## Introduction

The filarial worm diseases onchocerciasis (“river blindness”) and lymphatic filariasis (LF, “elephantiasis”), though not typically lethal, produce substantial morbidity, social stigma and loss of economic opportunity in tropical and subtropical regions throughout the globe [1,2]. Nearly 150 million people are currently infected with these parasites, with a greater number at risk; more than 40 million suffer from symptomatic disease. Current treatments for these “neglected tropical diseases” (NTD’s) typically involve periodic mass drug administration (MDA), with the goal of reducing disease prevalence and ideally triggering elimination. Populations in *Onchocerca-*endemic regions are administered an annual or semi-annual dose of ivermectin; LF-endemic communities normally receive a combination of albendazole with ivermectin in sub-Saharan Africa or with diethylcarbamazine elsewhere [3]. More recently, the World Health Organization (WHO) has explored the use of triple-therapy employing ivermectin, diethylcarbamazine and albendazole, recommending its use in specific settings [4]. Recently-approved moxidectin [5] may offer some advantage as a replacement for ivermectin with a more sustained response. These agents primarily kill first-stage larvae (microfilariae, mf) and temporarily sterilize adult worms, but do not clear the primary infection. Consequently, MDA must be repeated at regular intervals to successfully affect disease prevalence.

Agents that effectively kill adult worms could greatly speed efforts toward elimination of these diseases, and thus are a critical priority for new filariasis drug development. It would also be beneficial to replace ivermectin in significant portions of West Africa that are co-endemic for onchocerciasis and a third filarial disease, loiasis (caused by infection with *Loa loa* worms). Loiasis typically creates a high burden of circulating mf; treatment of co-infected individuals with ivermectin carries the risk of severe adverse effects or death [6]. An agent that selectively targets adult worms without acutely affecting mf would transform the treatment of these debilitating diseases.

Filarial worms causing onchocerciasis and LF carry an obligate symbiotic bacterium, *Wolbachia*, which is essential for worm fertility and ultimate survival. Clinical studies have demonstrated effective treatment of these diseases through depletion of *Wolbachia* by anti-bacterial therapy with doxycycline [7,8]. Of note, this mechanism has three distinct elements that are considered particularly desirable for a new anti-filarial agent:

1. It sterilizes adult worms rather than directly killing mf. Interrupting the production of mf results in the slow decline of circulating mf levels.
2. Adult worm death occurs slowly. After depletion of *Wolbachia*, adult worms are committed to death, but take months to be fully cleared from the host.
3. Microfilaria depleted of Wolbachia are less able to develop in the intermediate vector, and thus less competent to spread the disease [9]

Since the pathologies of both diseases have been associated with *Wolbachia* release, an agent that acts by reducing *Wolbachia* populations within the adult worm may have additional immunological benefits over agents that are directly macrofilaricidal. A slow-kill mode of action reduces the probability of adverse reactions related to sudden worm death.

The concept of targeting *Wolbachia* as an approach to treating filarial disease has been clinically validated using the tetracycline antibiotics doxycycline and minocycline [8]; however these drugs are not ideal for use in the field, as they are contraindicated in children and in women of child-bearing age. The long-term goal of the Anti-*Wolbachia* (A·WOL) Consortium is the discovery and development of novel anti-*Wolbachia* agents with superior profiles. Recently we reported the discovery of a new anti-*Wolbachia* compound, ABBV-4083, derived from the macrolide antibiotic Tylosin A [10]. ABBV-4083 exceeds the efficacy of doxycycline and meets many of the stated pre-clinical goals for a next-generation anti-filarial agent. Herein we describe the details of the discovery program leading to the identification of this novel anti-filarial agent.

## Methods

### Ethics statement

Animal experiments using *Litomosoides sigmodontis* were performed at the Institute for Medical Microbiology, Immunology and Parasitology of the University Hospital Bonn, Bonn, Germany, in accordance to the European Union animal welfare guidelines (Directive 2010/63/EU and the Amsterdam Treaty: Protocol on the protection and welfare of animals N°33) and all protocols were approved by the Landesamt für Natur, Umwelt und Verbraucherschutz, Cologne, Germany (AZ 84–02.04.2015.A507; 84–02.04.2012.A140). All pharmacokinetic studies were reviewed and approved by AbbVie's Lake County Institutional Animal Care and Use Committee. Animal studies were conducted in an AAALAC accredited program and veterinary care was available to ensure appropriate animal care.

### Preparation of Tylosin derivatives

**Fig 1:**
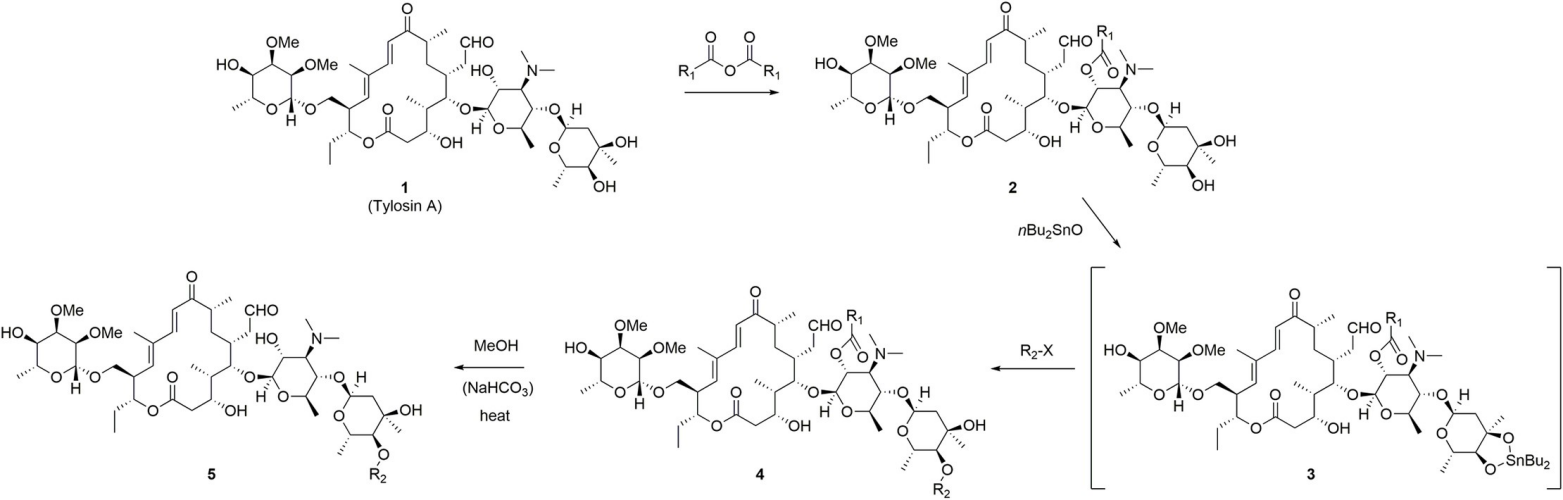
Preparation of Tylosin analogs.

Derivatives of Tylosin A were prepared from TylA (CAS 1401-69-0) or its L-(+)-tartrate salt (CAS 74610-55-2) using simple modifications of previously reported procedures, as illustrated in Fig 1. Selective acylation of the 2’-alcohol (on the mycaminose sugar) is accomplished under mild conditions employing acid anhydrides as reagents [11], presumably as a consequence of neighboring-group activation from the adjacent dimethylamino group [12]. Reaction with dibutyltin oxide forms a cyclic tin oxide between the vicinal diol pair at 3” and 4” (mycarose sugar); this serves as an activated intermediate for the selective acylation of the 4”-hydroxyl group [13]. Alkylation of this site is also possible, under more forcing conditions and using active alkylating agents.

When the 2’-substituent is acetyl, a free 2’-hydroxyl group may be liberated *via* heating in methanol. This transformation is accelerated through the addition of a small amount of solid NaHCO_3_. This straightforward sequence of transformations allows for the preparation of 2’-, 4”-, or 2’/4”-modified tylosin analogs, from common intermediates, in good overall yields (Table 1).

**Table 1:** Tylosin derivatives modified at 2’- and 4”-positions.

Paper ID	R_1_	R_2_
**1** (TylA)		
**2a**	CH_3_	
**2b**	CH(CH_3_)_2_	
**2c**	(CH_2_)_3_CH_3_	
**4a**	CH_3_	C(O)(CH_2_)_3_CH_3_
**4b**	CH_3_	C(O)NEt_2_
**4c**	CH_3_	CH_2_Ph(4-F)
**4d**	CH(CH_3_)_2_	C(O)NEt_2_
**4e**	CH(CH_3_)_2_	CH_2_Ph(4-F)
**5a**		C(O)(CH_2_)_3_CH_3_
**5b**		C(O)C(CH_3_)_3_
**5c**		C(O)NEt_2_
**5d**		CH_2_Ph(4-F)

### *In vitro* anti-*Wolbachia* cell based screening

Compounds were screened for anti-*Wolbachia* activity *in vitro* in the A·WOL-validated *Wolbachia*-infected *Aedes albopictus* (C6/36 *w*AlbB) 7-day cell-based assay which utilizes a 384-well format assay with high content imaging (HCI) (Operetta) as described previously [14].

### Pharmacokinetic studies

PO doses were administrated by oral gavage, IP doses by intraperitoneal injection to BALB/c mice or Sprague-Dawley rats (Charles River Laboratories, USA). Serial blood samples collected into EDTA anticoagulant for plasma concentration analysis were obtained from each animal after dosing. EDTA preserved plasma samples were extracted by protein precipitation with acetonitrile fortified with internal standards. The supernatant was injected into an HPLC-MS/MS system for separation and quantitation. Detection was accomplished using a triple quadrupole mass spectrometer operated either in electrospray or atmospheric chemical ionization (APCI) mode. The area under the plasma concentration-time curve (AUC) was calculated using the linear trapezoidal rule.

### *In vivo* screening, *Litomosoides sigmodontis* rodent model

Mice and jirds (*Meriones unguiculatus*, both obtained from Janvier, Saint-Berthevin, France) were kept in individually ventilated cages with food and water *ad libitum* and a light/dark cycle of 12h.

As described previously [15], female BALB/c mice or female jirds were infected at 6–8 weeks of age with *L*. *sigmodontis* larvae through the bites of *Ornithonyssus bacoti* mites. The same batch of L3 larvae-containing mites were used for all experimental groups within each experiment to ensure comparable rates of infection.

One day post infection, mice were dosed IP or PO with TylA (Sigma Aldrich, 200 mg/kg BID x 7 days), doxycycline hyclate (Sigma-Aldrich, 200 mg/kg BID x 14 days), or vehicle using a volume of 10 ml/kg. At 35 days post-infection mice were euthanized using an overdose of isoflurane; worms were recovered from the pleural cavity by pleural lavage, counted, sexed and staged for development into L4 and adult worms based on the difference of the buccal capsule through microscopic examination. Female worms were measured for length (mm) as a marker for development as previously described [16]. Data were distributed in a non-parametric fashion, median and interquartile ranges are presented. For comparing the length of the female worms, the Mann-Whitney-U test was used to calculate statistical differences either against the vehicle treated or gold standard groups.

Starting at 14 weeks post infection, microfilariae-positive jirds (n = 7 per group) were dosed PO with ABBV-4083 (150 mg/kg QD) dissolved in 0.5% HPMC/0.02% Tween-80 or vehicle using a volume of 5ml/kg. Microfilariae numbers were assessed through visual inspection of blood samples collected from the saphenous vein at weekly intervals post-dosing. For this, 10 μl of peripheral blood were diluted in 300 μl of Hinkelmann solution (0.5% Eosin Y, 0.5% Phenol, 0.185% Formaldehyde in aqua dest). After 5 minutes of centrifugation at 400g, the supernatant was discarded and the pellet transferred for microscopic quantification of the microfilariae. At 16 weeks post-treatment, jirds were euthanized by an overdose of isoflurane; worms were recovered from the pleural cavity and counted. Remaining intact female adult worms were used to assess embryogenesis and the *Wolbachia* load. For the latter, genomic DNA (gDNA) was extracted from individual female adult worms and quantification of the *Wolbachia ftsZ* (*wLs-ftsZ*) and *L*. *sigmodontis* β-actin (*Ls-act*) gene copy numbers was performed by quantitative real-time PCR (qPCR) [16].

For embryograms, remaining intact female adult worms were individually homogenized in 20% Hinkelmann/80% PBS solution, diluted 1:10 in PBS and quantified by microscopy. Embryonal stages were differentiated as egg, morulae, pretzel and stretched microfilariae [17].

## Results and discussion

### Identifying a novel lead

We began our work by selecting a diverse and representative sample of the AbbVie antibiotics collection (129 compounds) for single-point testing against *Wolbachia pipientis* in an insect cell line [14]. This screen revealed several novel leads, most notably the established veterinary antibiotic Tylosin A (**1**, Fig 1). While TylA has a long history of use in multiple animal species, it has never been studied in humans; and its activity against *Wolbachia* has not previously been reported. It is a potent anti-*Wolbachia* agent, with an EC_50_ value of 28 nM (measured in *Wolbachia*-infected insect cells as described above), similar to that for doxycycline. Other commercially available 16-membered macrolides (spiramycin, josmycin, midecamycin and leucomycin) are inactive against *Wolbachia*; similarly 58 semisynthetic leucomycin derivatives from the AbbVie collection showed no activity at 10 μM concentration. Notably, none of these macrolides contain the mycinose sugar present in TylA. In contrast, Tylosin B (TylB), which contains the mycinose residue but lacks the mycarose sugar of TylA, retained substantial though reduced activity against *Wolbachia in vitro* (EC_50_ 88 nM).

**Fig 2:**
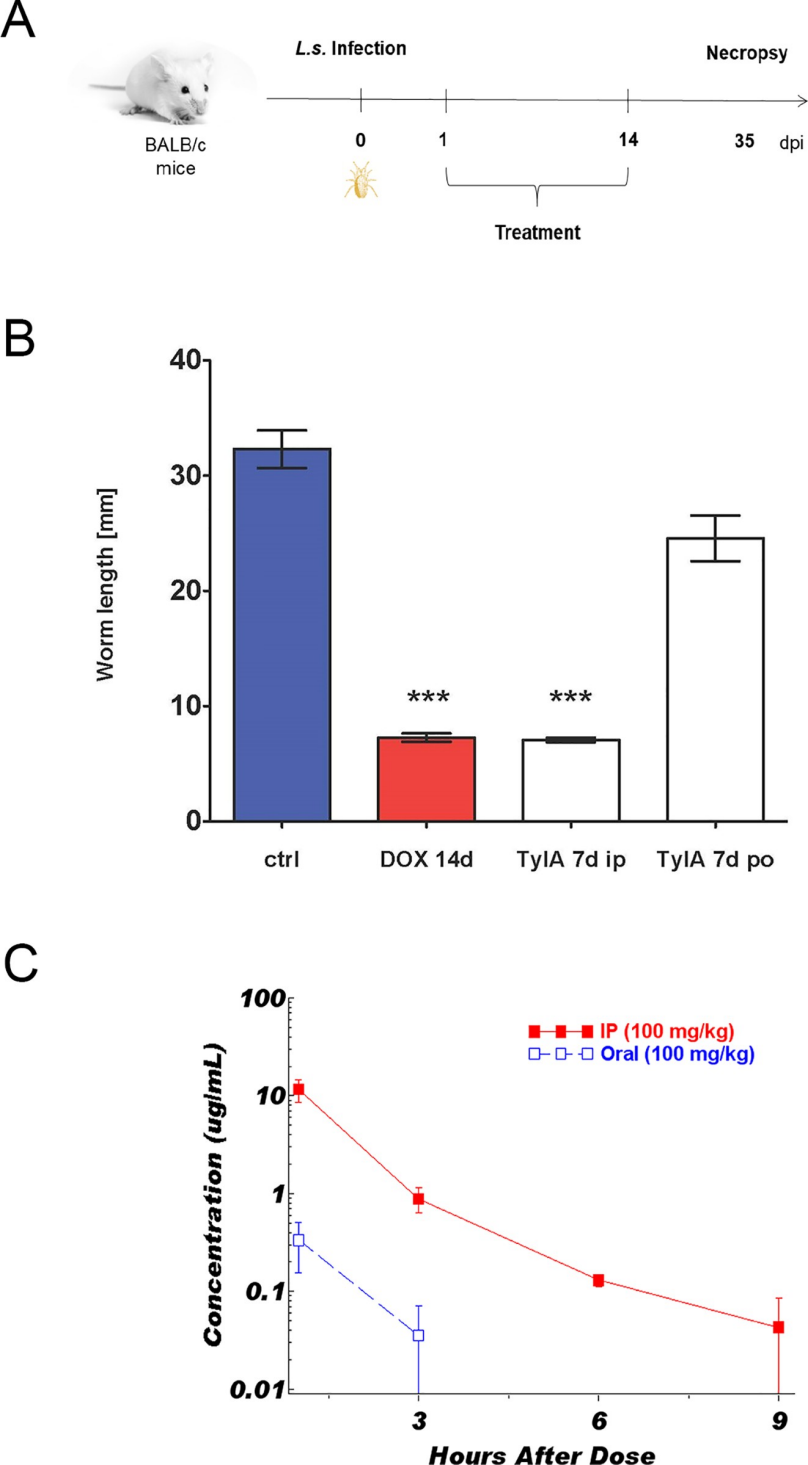
Poor oral bioavailability of Tylosin A impairs clearance of *Wolbachia* endosymbionts *in vivo*. **A**. Experimental design for *L*. *sigmodontis* larval mouse study. **B**. Worm lengths (an indicator of development) from *L*. *sigmodontis* larval mouse model; animals treated with doxycycline (200 mg/kg PO BID X 14 days) or TylA (200 mg/kg IP or PO, BID X 7 days) or vehicle control (VC). By simple non-parametric Mann-Whitney test: TylA IP against TylA PO and TylA vs vehicle are highly significant p<0.0001, TylA IP vs DOX control and vehicle vs TylA PO = ns **C**. Plasma levels of TylA in BALB/c mice following IP or PO dosing (100 mg/kg).

As follow-up to this initial *in vitro* study, we examined the activity of TylA in a mouse model of filarial disease [15]. Mice naturally-infected with *L*. *sigmodontis* through mite bites were treated with TylA or doxycycline at a dose of 200 mg/kg twice daily (Fig 2A). When TylA was dosed IP for 7 days, recovered worms were notably shorter than controls, indicating that development has been suppressed. This result is similar in magnitude to that produced by 14 days of doxycycline treatment (Fig 2B). Oral dosing of TylA, however, produced a minimal response. Supplementary experiments have correlated this growth stunting phenotype with a reduction in *Wolbachia* levels [10].

These results were readily explained by examination of circulating drug levels measured in a companion pharmacokinetic study (Fig 2C). Drug levels in the IP arm of this study were >30-fold higher than those achieved when the drug is given PO. We suspect that the poor oral bioavailability of TylA results from an inability to efficiently cross membranes like the gut lining; the compound exhibits very low permeabililty (<0.1X10^-6^ cm/sec) in a canine kidney cell monolayer system (MDR-MDCK). Therefore, improving drug absorption by increasing permeability became a primary goal for our lead-optimization studies.

### Lead optimization and candidate selection

We focused our initial Structure-activity relationship (SAR) studies on modifications that reduce the H-bond donor capacity of our lead; hypothesizing that the large number of free hydroxyl groups (TylA has 5 free–OH’s) was responsible for the poor permeability (and thus poor bioavailability) of TylA. The most readily accessible of these hydroxyl groups is the 2’-OH (on the mycaminose sugar), which is internally activated by an adjacent amine functionality. Thus, as previously reported by Tsuchiya and others, this position may be acylated under mild conditions [11,12]. Acylation of the 2’-position causes a modest but significant loss of potency against *Wolbachia*; esters **2a**-**2c** (Table 1) have EC_50_ values that are 2–3 fold higher than parent TylA (Table 2).

**Table 2:** *In vitro* activity (*Wolbachia* EC_50_) and drug exposure levels (rat pharmacokinetic studies) for Tylosin analogs. Representative TylA analogs were evaluated for anti-*Wolbachia* activity using a high-content imaging system in insect cells; replicate experiments were averaged to determine an EC_50_ value for each compound (column 2). A subset of analogs were dosed PO to Sprague-Dawley rats, with plasma drug levels recorded at regular intervals to determine total AUC of parent drug (column 3) and of the primary metabolite TylA (column 4). Potency-weighted AUC (column 5) and time-over-EC_50_ (column 7; measured as EC_50_-multiple at 8 hrs) are taken as predictors of *in vivo* activity, and were used to prioritize compounds for further study.

Compound #	*Wolbachia* EC_50_ (N)	AUC* parent	AUC* TylA (metabolite)	AUC*/EC_50_	C*_8hr_	C*_8hr_/ EC_50_
**1** (TylA)	28 nM (5)	9.6	N/A	0.34	0.5	0.02
**2a**	90 nM (1)	ND	ND	—	ND	—
**2b**	78 nM (2)	ND	ND	—	ND	—
**2c**	65 nM (2)	58	1.2	0.89	2.5	0.04
**4a**	6.1 nM (3)	ND	ND	—	ND	—
**4b**	6.6 nM (2)	ND	ND	—	ND	—
**4c**	2.4 nM (4)	ND	ND	—	ND	—
**4d**	24 nM (2)	420	1.4	17.5	30	1.25
**4e**	29 nM (2)	510	<0.6	17.6	30	1.03
**5a**	1.3 nM (3)	<0.6	30	N/A	N/A	—
**5b**	5.4 nM (4)	58	<0.6	10.7	3.0	0.56
**5c**	1.3 nM (4)	40	1.0	30.8	1.5	1.15
**5d**	0.019 nM (4)	16	<0.6	842	0.75	39.5

EC_50_ determined as geometric mean (N), each N a duplicate measurement

AUC* = AUC/dose; units ng-hr/ml per mg/kg

AUC*/EC_50_, units ng-hr/ml/nM per mg/kg

C*(8hr) = C(8hr)/dose; units ng/mL per mg/kg

C*(8hr)/EC_50_, units ng/ml/nM per mg/kg

To test our hypothesis regarding the role of the free hydroxyl groups in impeding the uptake of TylA, we compared the pharmacokinetic profiles of **2c** and TylA in rodents (Table 2). Acylation of the 2’-position leads to a 6-fold improvement in plasma drug levels (as expressed by total area-under-the-drug-exposure-curve, AUC). In the study of compound **2c** we also looked for the presence of TylA (the de-acylated metabolite), determining that an ester group at this position is relatively metabolically stable. This improvement in drug exposure is enough to override the modest loss of potency that comes with acylation; the potency-weighted AUC (determined as AUC/EC_50_) is ~2.6 times higher with the 2’-valerate ester, and the potency-weighted 8-hr drug level (determined as C_8hr_/dose/EC_50_) doubles. We have previously noted that maintaining free drug levels above the EC_50_ value is an important determinant of *in vivo* efficacy [10].

This early result seems to support our central hypothesis, and encouraged us to explore the effect of modifying other hydroxyl groups in TylA. We had notable success through modification of the 4”-OH, on the mycarose sugar. Once the 2’-OH has been derivatized, selective activation of the 4”-position is possible through formation and acylation of a 3”/4”-cyclic tin complex (e.g. compound **3a**/**b**, Fig 1), as previously described by Kiyoshima *et al*. [13] When the 2’-substituent is an acetyl group, the corresponding 2’/4”-diacylated analog **4a** (Table 1) may be selectively deacylated at the 2’-position simply by warming in methanol, to give the 4”-mono-ester **5a**. Unexpectedly, modification of this 4”-site significantly improved the activity of the resultant derivatives against *Wolbachia*; for example, ester **5a** has an *in vitro* EC_50_ of 1.3 nM (Table 2), 25-fold lower than TylA.

The pharmacokinetic profile of compound **5a** was examined in mice. As with the previous study of 2’-ester **2c**, we observed that oral drug levels increase (~3-fold) upon 4”-acylation (Table 2). However, in this case the primary drug measured in the plasma is not the parent ester; rather, it is the deacylated metabolite **1** (TylA). In fact, no sign of parent is observed at any time point in this study, suggesting a very rapid cleavage of the 4”-ester moiety. The metabolic susceptibility of these 4”-esters (presumably to hepatic esterases, though this has not been proven) is substantially higher than that of the corresponding 2’-esters, though the latter are more susceptible to chemical hydrolysis.

While it is possible that this metabolic pathway is rodent-specific, the result suggested to us that another solution was desirable. To this end, we explored several strategies for modifying the 4”-position with substituents expected to have greater metabolic stability.

#### Hindered esters

Metabolic processing of the 4”-valerate ester **5a** is quite rapid, despite the relatively high level of steric hindrance on the O-side of this ester linkage. We speculated that an increase in steric bulk on the carbonyl-side might help to suppress esterase processing. To this end we prepared the corresponding pivalate ester **5b** (Table 1). As with compound **5a**, this modification improves *in vitro* potency (EC_50_ = 5.4 nM, a 5X-improvement over TylA; see Table 2); and analogous to compound **5a**, it leads to an improvement in oral absorption (AUC* = dose-weighted AUC = 55 ng-hr/ml per mg/kg, a 6-fold improvement over TylA). In this case, however, essentially all of the measured drug is the active parent; <1% of the deacylated product is noted during a rat pharmacokinetic study. When the potency and absorption gains are factored together (by determining a potency-weighted AUC* = AUC*/EC_50_), pivalate ester **5b** is 30-fold superior to TylA as an oral anti-*Wolbachia* agent. A similar increase in potency-weighted 8-hr drug levels is observed.

#### Carbamates

Carbamates are structurally similar to esters, but are generally not susceptible to the action of esterases. Reaction of tin reagent **3** with carbamyl chlorides provides carbamates like **4b** (Table 1), though this transformation generally requires longer reaction times and/or higher temperatures than the corresponding acylations. Methanolysis of the 2’-acetate **4b** gives the corresponding **5c**. The carbamate modification of the 4”-OH is very well tolerated; compound **5c** has a *Wolbachia* EC_50_ of 1.3 nM, a >20X potency improvement over parent TylA (Table 2). Pharmacokinetic evaluation in rats demonstrates an absorption/elimination profile similar to that of the 4”-pivalate **5b**; the dose-weighted AUC is 40 ng-hr/ml per mg/kg, less than that of **5b** but still 4X-higher than that of TylA. Notably, the carbamate group is also metabolically stable; a small amount of TylA is measured in the circulation when **5c** is dosed orally, but it is <3% of the total circulating drug. Combining the potency and pharmacokinetic gains, carbamate **5c** is 90-fold superior to TylA in rodents on the basis of potency-weighted AUC, and ~60-fold improved with regard to potency-weighted 8-hr drug levels.

#### Benzyl ethers

We anticipated that we could completely suppress esterase cleavage at the 4”-position through elimination of the relevant carbonyl group, i.e. by preparing 4”-ethers. In practice 4”-alkylation is a slow process; only very reactive electrophiles (e.g. methyl iodide and benzyl halides) react with tin reagents like **3**. Using more stringent conditions, benzyl ethers like **4c** could be prepared, then methanolized to give **5d** (Table 1). Compound **5d** represents an extreme example of the *in vitro* potency benefit that may be gained through modification of the 4”-position; this analog has a *Wolbachia* EC_50_ of 0.019 nM, a 1,500-fold improvement over parent TylA (Table 2). A rat pharmacokinetic study of this compound confirms the esterase stability of the ether linkage; only parent drug is observed in the plasma. Compared with the previous examples, the benzyl ether provides only a modest improvement in circulating drug levels (1.7 fold); however the large potency boost leads to a dramatic increase in potency-weighted AUC. AUC*/EC_50_ for **5d** is 842,000 ng-hr/ml/uM per mg/kg, a 2,500-fold improvement over our original lead; and C*_8hr_/EC_50_ has increased 2,000-fold.

This potency boost observed upon 4”-modification seems to be *Wolbachia*-specific. When **5d** is profiled against a panel of gram(+) and gram(-) microorganisms, the majority show little to no change in susceptibility (as compared with TylA) upon addition of this substituent [10].

#### 2’/4”-Modified analogs

Since derivatization of either the 2”- or 4”-hydroxyl group in TylA provides a pharmacokinetic benefit, we were curious to explore whether a combination of these features might produce an even more robust drug-exposure profile. To this end we prepared compounds **4d** and **4e**, analogs of **5c**/**5d** which also contain the 2’-isobutyrate ester of **2b**. In fact, this combination of modifications does provide a notable pharmacokinetic benefit; oral dosing of **4d** and **4e** provide drug levels (in rats) that are more than 10-fold higher than those of their 2’-OH partners **5c** and **5d**, and 40–50 times higher than that of TylA itself (Table 2). These gains, though, come at a cost of *in vitro* potency. It seems that the presence of the 2’-ester suppresses the potency gain achieved through 4”-modification; both doubly-modified derivatives have anti-*Wolbachia* potencies in the range of TylA and **2b**. In the end, this potency loss overwhelms the pharmacokinetic gains; though **4d** and **4e** are superior to TylA (in terms of potency-weighted AUC* and C*_8h_) they are noticeably inferior to **5c** and **5d**.

From these initial results, **5c** and **5d** were selected for extensive *in vivo* characterization. Briefly, in a variety of efficacy studies in three species of adult filarial worms (*Litomosoides*, *Brugia* and *Onchocerca*) [10], potent anti-*Wolbachia* activity superior to doxycycline with shorter durations of treatment is observed in these models, along with resulting disrupted embryogenesis within adult female worms. As a consequence of these studies, compound **5d** (designated ABBV-4083) was selected as a candidate for further evaluation.

### *In vivo* pharmacology

**Fig 3:**
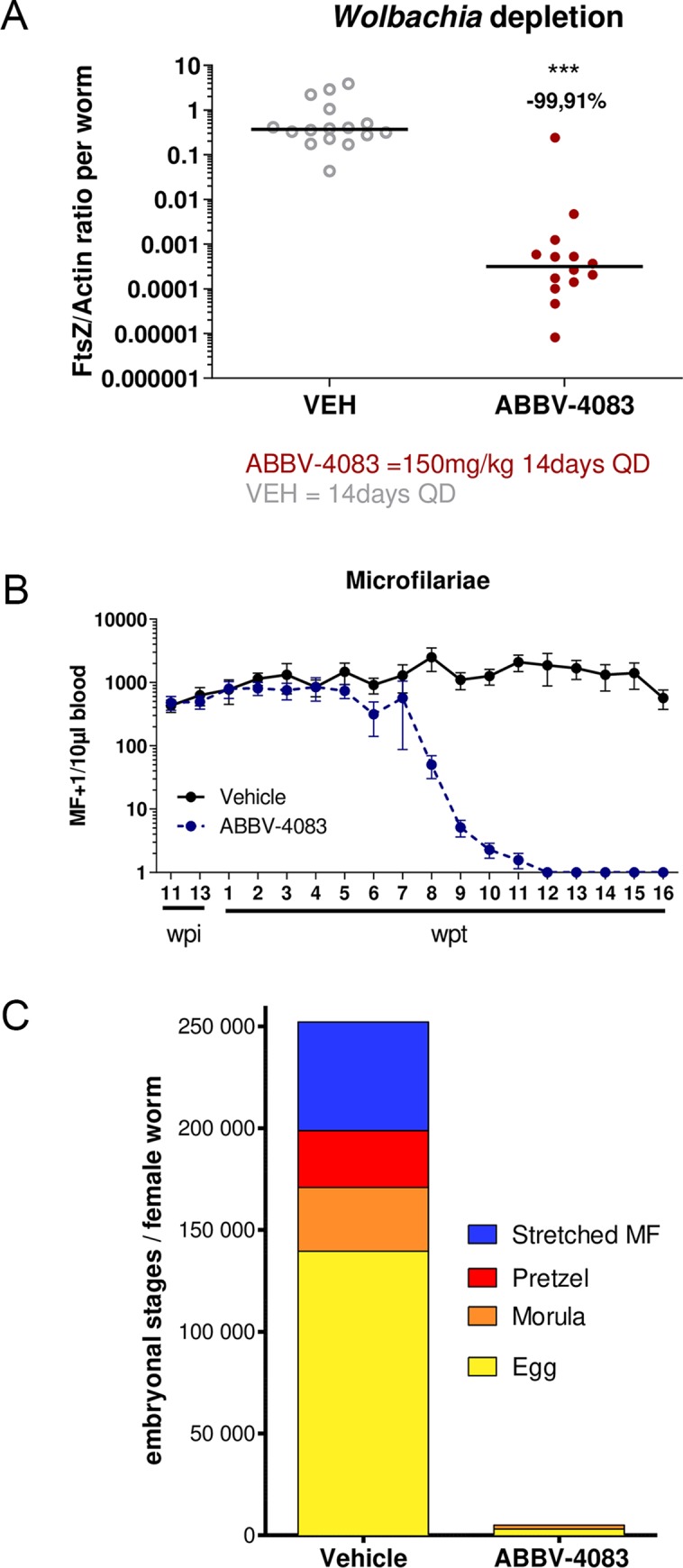
Oral ABBV-4083 treatment in jirds reduces *Wolbachia* levels in *L*. *sigmodontis*, clears microfilaremia and blocks embryogenesis. Microfilariae-positive jirds were treated with 150 mg/kg PO ABBV-4083 (n = 7) or vehicle control (n = 7) for 14 days. **A**, *Wolbachia* levels in recovered *L*. *sigmodontis* female adult worms measured 16 weeks post-treatment start (vehicle n = 16; ABBV-4083 n = 14) and **B**, circulating levels of *L*. *sigmodontis* microfilariae (MF) in 10μl of peripheral blood. **C**, embryograms from female adult worms isolated at 16 weeks post treatment start (vehicle n = 3; ABBV-4083 n = 6) showing the median number of eggs, morulae, pretzel and stretched MF within *L*. *sigmodontis* uteri.

The following representative experiment is illustrative of the *in vivo* activity of ABBV-4083. *Litomosoides sigmodontis* is a filarial parasite that leads to patent infections in BALB/c mice and gerbils (jirds); natural infection can be established through mites [15]. When infected jirds (*Meriones unguiculatus*) are treated with ABBV-4083 at an oral dose of 150 mg/kg, once daily for 14 days, *Wolbachia* levels (measured 16 weeks post-treatment-initiation, pti) are reduced by >99.9% in the recovered female adult worms (Fig 3A). As predicted, these reductions in symbiont levels had consequences for worm fertility. Starting at ~7 weeks pti, levels of circulating microfilariae declined (Fig 3B) and were completely cleared from 12 weeks pti until the end of this study at 16 weeks pti. Control animals maintained circulating levels of microfilariae throughout the study. We have previously demonstrated that ABBV-4083 is not directly microfilaricidal [10], so it is likely that this decrease is a consequence of a loss of worm fertility. In fact, analysis of the uterine contents of female worms (“embryograms”) indicate a profound effect on embryogenesis (Fig 3C), as suggested by the near-complete loss of all embryonic forms including eggs. Additional experiments [10] demonstrate that ABBV-4083 equals or exceeds the efficacy of doxycycline with regard to *Wolbachia* depletion and maintenance of microfilariae clearance even when the latter is dosed for substantially longer intervals (e.g. 14- vs 7-days), strongly suggesting the possibility that ABBV-4083 might provide a shorter-course treatment for filarial diseases.

### Safety assessment

As a preclinical candidate, ABBV-4083 has been evaluated in a variety of *in vitro* assays assessing preclinical safety. In an initial battery of 35 assays assessing the general selectivity of the compound, there were no significant interactions with any receptors at a maximum concentration of 10 μM. This pattern was confirmed in studies across 77 mammalian receptors, ion channels, enzymes and transporter assays, in which a significant interaction was only observed in two assays [10]. ABBV-4083 did not inhibit functional hERG channel activity at a maximum concentration of 30 μM, and did not produce significant cardiovascular effects when administered to dogs. The compound was neither mutagenic nor clastogenic in *in vitro* genotoxicity screening assays. No potential to induce phospholipidosis was observed *in vitro*, and the compound did not induce steatosis in an *in vitro* high-content screen. In preparation for first-in-human studies, the safety of ABBV-4083 has been extensively evaluated in 28-day GLP general and reproductive toxicity studies. In addition, the synthesis has been adapted to produce GMP quality supplies.

### Conclusions

Through properties-driven optimization of the anti-*Wolbachia* lead Tylosin A, we have identified ABBV-4083, an analog with a superior pharmacokinetic profile and remarkably improved potency. This combination of improved properties addresses the liabilities of TylA itself, and the analog appears suitable for use as an oral therapeutic for treating onchocerciasis and/or lymphatic filariasis. Based on preclinical data, ABBV-4083 demonstrates potential improvements over the use of doxycycline as an anti-*Wolbachia* agent in terms of both safety and reduced treatment duration. Given the short synthesis of this compound from a widely available and inexpensive veterinary product, its use for neglected diseases such as onchocerciasis and lymphatic filariasis should not be limited by cost of goods. Whether ABBV-4083 is best suited for MDA or test-and-treat strategies will only become evident after clinical trials defining its efficacy and safety. Phase 1 studies of this agent in normal healthy human volunteers are currently underway; results will be reported in due course.
